# Ophthalmic findings of congenital insensitivity to pain with anhidrosis with a novel neurotrophic tyrosine kinase receptor type 1 gene mutation: A case report

**DOI:** 10.3389/fmed.2022.955929

**Published:** 2022-09-07

**Authors:** Rong Zhu, Yuxiang Zhu, Mingpeng Xu, Zhensheng Gu

**Affiliations:** Department of Ophthalmology, Xinhua Hospital, Affiliated to Shanghai Jiao Tong University School of Medicine, Shanghai, China

**Keywords:** congenital insensitivity to pain with anhidrosis (CIPA), *NTRK1*, cornea, neurotrophic keratopathy, dry eye

## Abstract

We report a case of congenital insensitivity to pain with anhidrosis (CIPA) with a novel neurotrophic tyrosine kinase receptor type 1 (*NTRK1*) gene mutation. The patient suffered from recurrent corneal ulcer. A slit-lamp examination revealed ciliary hyperemia, bulbar conjunctival edema, epithelial defect, and ulcer lesion in the inferior part of the cornea, local corneal stromal edema accompanied by new vascular growth in his affected eye. In addition, the corneal sensitivity and nerve fiber density decreased significantly in both eyes. Tear film break-up time and Schirmer’s I test were below lower limit. Moreover, the patient exhibited typical systemic features, including no normal response to pain stimuli, anhidrosis and self-injurious behavior. Gene sequencing revealed a compound-heterozygous mutations in *NTRK1* gene: a missense mutation inherited from his mother (c.1750G > A, P.E584K) and a new splicing mutation inherited from his father (c.2187 + 5G > C). After 8 weeks of medication, the corneal ulcer basically healed. This study expands the spectrum of *NTRK1* gene mutation associated with CIPA and provides a feasible approach for clinicians to treat patients with CIPA-related keratopathy.

## Introduction

Congenital insensitivity to pain with anhidrosis (CIPA), also known as hereditary sensory and autonomic neuropathy type IV, is a rare autosomal recessive disorder caused by mutations in the *NTRK1* gene on chromosome 1q23 (1). First reported by Swanson in 1963, CIPA is characterized by lack of normal responses to pain stimuli, anhidrosis (inability to sweat), recurrent episodes hyperpyrexia, self-injurious behavior, and mild to severe intellectual disabilities (2, 3). Herein, we report a case of ophthalmic and systemic manifestations in a Chinese patient with genetically confirmed CIPA and identify a novel mutation of the *NTRK1* gene.

## Case presentation

**FIGURE 1:**
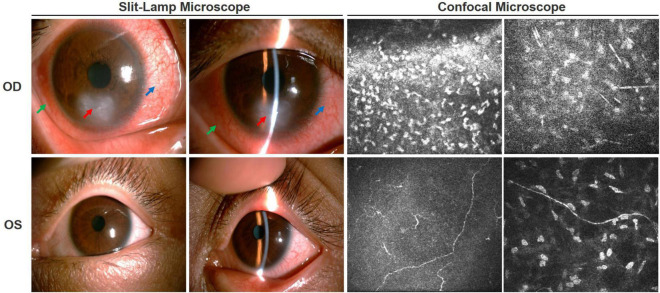
Ocular condition of both eyes before treatment. Slit-lamp examination showed ciliary hyperemia of the right eye (blue arrow), bulbar conjunctival edema (green arrow), epithelial defect, ulcer lesion (red arrow), and local corneal stromal edema accompanied by new vascular growth. There was no obvious abnormality in the left eye. Corneal confocal microscopy showed a large number of inflammatory cells infiltrated in the ulcer area of the right eye, corneal tissue structure disordered, and nerve fibers became sparse and arranged chaotically. In the left eye, the density of the subepithelial nerve fiber plexus was significantly decreased or even vanished, some of which ruptured and twisted.

A 30-year-old man with recurrent ocular hyperemia in his right eye was diagnosed with viral keratitis at his local hospital and treated continuously with antiviral eye drops. His condition subsequently worsened, and he was referred to our hospital for further evaluation. A comprehensive ophthalmic examination was performed during his first visit, the uncorrected visual acuity was 20/50 in both eyes, and the intraocular pressure was 14.3 mmHg in the right eye (Oculus Dexter, OD) and 16.7 mm Hg in the left eye (Oculus Sinister, OS). There was obvious ciliary hyperemia in the right eye with bulbar conjunctival edema, a gray and white flake ulcer lesion in the inferior part of the cornea, an epithelial defect in the ulcer lesion, and local corneal stromal edema accompanied by new vascular growth. No obvious corneal abnormality was observed in the left eye (Figure 1). There were no complaints of dryness or eye pain. His parents revealed that he had not responded to pain stimuli from an early age, exhibited self-injurious behaviors, such as tongue biting, nail pulling, and did not sweat, even in the hot summer, and had recurrent episodes of elevated body temperature. Based on the patient’s ocular and systemic manifestations, we initially suspected that the patient might have congenital hereditary sensory and autonomic neuropathy, resulting in damage to the trigeminal nerve that innervates the cornea, weakening or even leading to the disappearance of corneal sensation, giving rise to dry eye and eventual ulceration.

**FIGURE 2:**
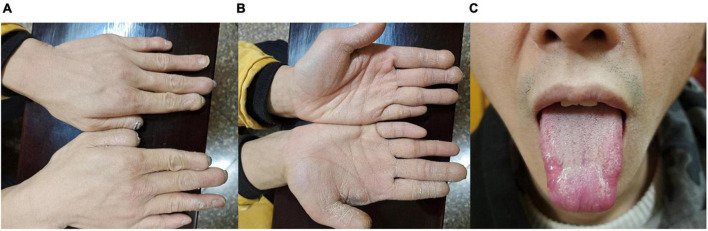
Systemic manifestations. Clinical symptoms of the patient: Spontaneous amputation of fingertips **(A)**, dry and hyperkeratotic skin of the palm **(B)**, damaged tongue **(C)**.

To clarify the etiology, we performed a detailed history and thorough clinical examination. The patient’s mother had spontaneous vaginal birth at term, prenatal examination did not detect any abnormalities. The patient’s parents were physical healthy and not consanguineous. Physical examination showed that the patient had microcephaly, low ears, sparse eyebrows, tongue tip scar formation, extremely dry palm skin, and hyperkeratosis, spontaneous amputation of fingertips on both hands (Figure 2). Additionally, he is unable to sweat anywhere on his body. Sensory examination showed no response to any pain stimuli or insensitivity to thermal sensation, while vibration and subtle touch still existed. Motor nerve conduction test showed the left peroneal nerve compound muscle action potential amplitude was reduced, while the somatosensory evoked potentials of four limbs were normal. His general intelligence and MRI of the brain were normal. Ophthalmological examination revealed that his corneal reflex had disappeared. The corneal sensitivity was measured using Cochet-Bonnet aesthesiometers at five locations of the cornea (central, superior, inferior, nasal, and temporal). The measurements of the right eye in the central and inferior cornea were 0 mm, in other locations were 5 mm, and 20 mm in the five locations of the left eye. Generally, less 40 mm is considered abnormal corneal sensation. The results showed that his corneal sensation was significantly decreased in both eyes. His tear film break-up time was 3.25 s (OD) and 4.08 s (OS). The Schirmer’s I test was 5 mm/5 min (OD) and 4 mm/5 min (OS), both of which were below the lower limit, indicating a reduction of reflex tear secretion. Corneal confocal microscopy showed a large number of hyperreflective inflammatory cells infiltrated in the ulcer area of the right eye, the corneal tissue structure was disordered, and the nerve fibers in the stromal layer became sparse and arranged chaotically. The density of the subepithelial nerve fiber plexus of the left eye was significantly decreased or even vanished, some of which ruptured and twisted (Figure 1). The clinical manifestations and complications of our patient were the same as those observed in patients previously reported in the literature.

**FIGURE 3:**
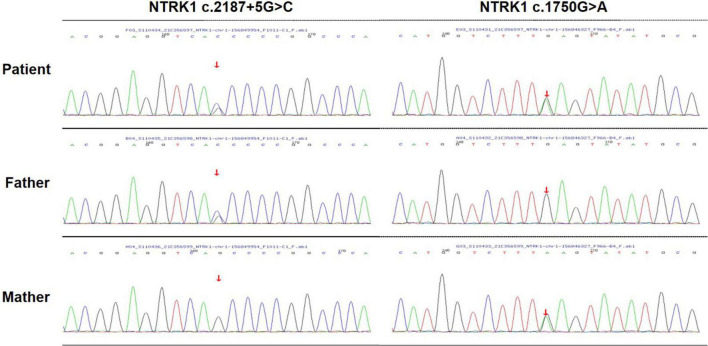
Gene sequencing. Sequencing analysis of the patient revealed a compound-heterozygous mutation in *NTRK1*.

The peripheral blood samples of patients and their families were collected for gene detection to confirm clinical diagnosis. The DNA sample of the patient was subjected to Targeted next-generation sequencing (NGS) on a panel of 210 genes, which were associated with peripheral neuropathy. The family members were confirmed by Sanger sequencing. The compound-heterozygous mutations were identified in NTRK1 gene of the patient’s: a missense mutation (c.1750G > A, p.E584K) on one allele, and a splicing mutation (c.2187 + 5G > C) on the other allele. The two identified mutations occurred in exons 13 and 15 of *NTRK1* gene, respectively. In addition, *NTRK1* c.2187 + 5G > C mutation was a novel mutation, which has not been reported before. Sanger sequencing showed that the patient’s mother carried the missense mutation, while his father carried the splicing mutation (Figure 3). The results indicated autosomal recessive *NTRK1*-related CIPA.

## Treatment

The patient was given levofloxacin eye drops twice a day to prevent bacterial infection, tacrolimus eye drops three times a day to control inflammation, and calf serum extract protein-free eye gel four times a day to promote ulcer repair. Additionally, the coenzyme Q10 capsules 20 mg were given orally three times a day to nourish nerve.

## Outcome and follow-up

**FIGURE 4:**
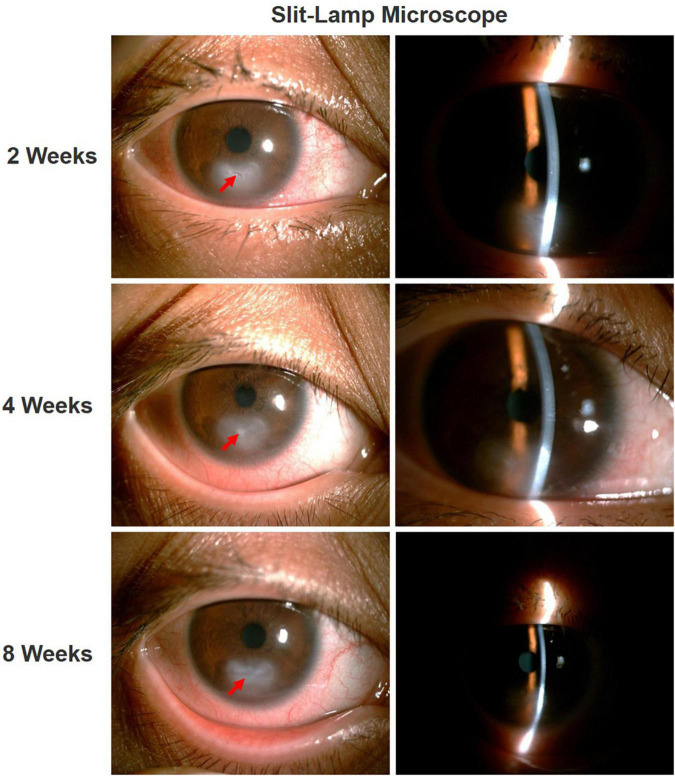
Ocular condition of right eye after treatment. After treatment, the ciliary hyperemia of the right eye was significantly reduced, the corneal ulcer gradually healed (red arrow), with residual corneal stromal opacification.

After 2 weeks of treatment, the ciliary hyperemia of the right eye was significantly relieved, and corneal ulcer underwent a slight improvement. After 4 weeks of treatment, the uncorrected visual acuity of the patient’s right eye was still 20/50, and the decreased corneal sensation did not improve, but corneal ulcer was partial repaired, corneal neovascularization significantly decreased, and corneal opacity was observed. Thereafter, the patient was instructed to continue treatment with reduced doses of calf serum extract protein-free eye gel and tacrolimus for more weeks. After 8 weeks of treatment, the uncorrected visual acuity of the right eye improved to 20/40, the ulcer lesion almost completely healed, and there was corneal stroma scar formation, the Schirmer’s I test results were still at the lower limit (less than 5 mm/5 min). Through the combination of the above drugs, the patient’s right eye lesion being well controlled by the time of the last follow-up exam (Figure 4).

## Discussion

CIPA mainly affects peripheral sensory and autonomic neurons, sensory nerve dysfunction leads to chronic ulcers of the hands and feet, corneal ulcer, and arthropathy. At the same time, insensitivity to pain and thermal sensations could lead to multiple fractures, burns and sometimes self-mutilation of fingers, tongue, and lips. Autonomic dysfunction leads to manifestations like anhidrosis and fever (4, 5). In recent years, with the accumulation of clinical experience and the development of genome sequencing technology, it has been found that the recessive mutation of *NTRK1* may lead to CIPA. So far, according to the Human Gene Mutation Database, 128 *NTRK1* gene mutations have been reported in CIPA patients and families (6).

The human *NTRK1* gene encodes neurotrophic tyrosine kinase, receptor, type 1, also called tropomyosin-related kinase A (TRKA), which contains 17 exons located on chromosome 1q23 and is the preferred receptor for nerve growth factor (NGF) (7). The TRKA-NGF system mainly controls neuronal differentiation signaling pathways, including neurite growth, neuronal survival and growth, and synaptic plasticity (8, 9). *NTRK1* mutations impair the structure and/or expression of TRKA protein, thereby, affecting NGF signal transduction, resulting in a lack of NGF-dependent neurons in CIPA patients, including nociceptive primary afferent sensory neurons and sympathetic postganglionic neurons (8, 10). Therefore, the patient showed completely insensitive to superficial and deep pain stimuli, absence of sweating and recurrent episodes of fever. We identified two heterozygous mutations (c.1750G > A, p.E584K and c.2187 + 5G > C) in *NTRK1* gene at exon 13 and 15, respectively. The mutation c.1750G > A (p.E584K) is a missense mutation inherited from his mother, this results in the conversion of Amino acid no. 584 from glutamate to lysine, which has been first reported by Geng et al. (2). The c.2187 + 5G > C mutation is a novel splicing mutation, inherited from his father, its clinical significance is uncertain at present. Studies have shown that exons 13–17 of *NTRK1* gene are responsible for encode the intracellular tyrosine kinase domain of TRKA protein, which is crucial for signal transduction, and mutations in this region may result in the production of aberrant proteins that cannot be activated and fail to transmit important signals for neuronal growth and survival (7).

Some CIPA patients first present at an eye clinic due to signs and symptoms of neurotrophic keratopathy (or keratitis). Differential diagnosis between the neurotrophic keratitis and the other keratitis should be paid attention, a detailed history and general examination can aid the diagnosis and treatment (11). Our patient presented with recurrent corneal ulcer. Because of the characteristic pain insensitivity and anhidrosis we were able to focus on the diagnosis based on clinical features (12). Affected Individuals with CIPA often have neurotrophic cornea injury in both eyes, which are caused by trigeminal nerve damage and reduced tear secretion (13). This decreased corneal perception puts the cornea at continuous risk of punctured keratitis, persistent epithelial defects, corneal ulcers, and even stromal melting (14). Therefore, the patient’s eye therapy aims to care for the dry eye, avoid additional corneal damage, prevent corneal infection and promote corneal healing. Hereby, closely following up during the treatment and adjusting the dosage in time are necessary.

Currently, the treatment of CIPA is limited to symptomatic management of the severe complications. Due to the lack of pain perception, children are vulnerable to external and self-damaging, so parents need to pay special attention to help children reduce the occurrence of accidents to some extent, such as daily inspection for injuries, home modification to prevent injury, wear anti-bite abrasive tools for teeth and so on. Additionally, due to the lack of sweating, patients are prone to severe hyperpyrexia, so special attention should be paid to the temperature regulation of the living environment. At the same time, apply moisturizer daily to protect the skin and use artificial tears to protect the eye surface. Finally, in families detected to have NTRK1 mutations, prenatal genetic testing and genetic counseling can be considered.

In summary, we reported a novel splice site mutation (c.2187 + 5G > C) in NTRK1 gene associated with CIPA. The present report expands the mutation spectrum of NTRK1 and further understands ophthalmic characteristics of CIPA, which will help facilitate future genotype–phenotype association studies.

## Ethics statement

Written informed consent was obtained from the individual(s) for the publication of any potentially identifiable images or data included in this article.

## Author contributions

RZ performed the data analyses and wrote the manuscript. YZ and MX helped perform data collection. ZG approved the final version. All authors contributed to the article and approved the submitted version.

## Supplementary material

Supplementary Figure 1 The timeline of thepatient.

## References

[1] Echaniz-Laguna A Altuzarra C Verloes A De La Banda MGG Quijano-Roy S Tudorache RA NTRK1 gene-related congenital insensitivity to pain with anhidrosis: a nationwide multicenter retrospective study. *Neurogenetics.* (2021) 22:333–41. 10.1007/s10048-021-00668-z 34405299

[2] Geng X Liu Y Ren X Guan Y Wang Y Mao B Novel NTRK1 mutations in Chinese patients with congenital insensitivity to pain with anhidrosis. *Mol Pain.* (2018) 14:1744806918781140. 10.1177/1744806918781140 29770739 PMC6009080

[3] Swanson AG. Congenital insensitivity to pain with anhydrosis. A unique syndrome in two male siblings. *Arch Neurol.* (1963) 8:299–306. 10.1001/archneur.1963.00460030083008 13979626

[4] Mantelli F Nardella C Tiberi E Sacchetti M Bruscolini A Lambiase A. Congenital corneal anesthesia and neurotrophic keratitis: diagnosis and management. *Biomed Res Int.* (2015) 2015:805876. 10.1155/2015/805876 26451380 PMC4588028

[5] Lopez-Cortes A Zambrano AK Guevara-Ramirez P Echeverria BA Guerrero S Cabascango E Clinical, genomics and networking analyses of a high-altitude native American Ecuadorian patient with congenital insensitivity to pain with anhidrosis: a case report. *BMC Med Genomics.* (2020) 13:113. 10.1186/s12920-020-00764-3 32807182 PMC7437939

[6] Li L Jia C Tang Y Kong Y Xia Y Ma L. Novel gross deletion mutations in NTRK1 gene associated with congenital insensitivity to pain with anhidrosis. *Front Pediatr.* (2021) 9:638190. 10.3389/fped.2021.638190 33748046 PMC7969531

[7] Luberg K Park R Aleksejeva E Timmusk T. Novel transcripts reveal a complex structure of the human TRKA gene and imply the presence of multiple protein isoforms. *BMC Neurosci.* (2015) 16:78. 10.1186/s12868-015-0215-x 26581861 PMC4652384

[8] Indo Y. NGF-dependent neurons and neurobiology of emotions and feelings: lessons from congenital insensitivity to pain with anhidrosis. *Neurosci Biobehav Rev.* (2018) 87:1–16. 10.1016/j.neubiorev.2018.01.013 29407522

[9] Rotthier A Baets J Timmerman V Janssens K. Mechanisms of disease in hereditary sensory and autonomic neuropathies. *Nat Rev Neurol.* (2012) 8:73–85. 10.1038/nrneurol.2011.227 22270030

[10] Li Y Tang X Yu X. Congenital insensitivity to pain with anhidrosis: a report of two unrelated Chinese families with novel mutations in NTRK1 gene. *Med Clin.* (2021) 157:451–3. 10.1016/j.medcli.2020.11.019 33422294

[11] Sethi A Ramasubramanian S Swaminathan M. The painless eye: neurotrophic keratitis in a child suffering from hereditary sensory autonomic neuropathy type IV. *Indian J Ophthalmol.* (2020) 68:2270–2. 10.4103/ijo.IJO_2101_19 32971688 PMC7728043

[12] Scanzera AC Shorter E. Case series. management of neurotrophic keratitis from familial dysautonomia. *Optom Vis Sci.* (2018) 95:678–81. 10.1097/OPX.0000000000001255 30063663 PMC8516073

[13] Semeraro F Forbice E Romano V Angi M Romano MR Filippelli ME Neurotrophic keratitis. *Ophthalmologica.* (2014) 231:191–7. 10.1159/000354380 24107451

[14] Axelrod FB Rolnitzky L Gold von Simson G Berlin D Kaufmann H. A rating scale for the functional assessment of patients with familial dysautonomia (Riley Day syndrome). *J Pediatr.* (2012) 161:1160–5. 10.1016/j.jpeds.2012.05.038 22727867 PMC3534733

