# Supplementary figures and images for: Ophthalmic findings of congenital insensitivity to pain with anhidrosis with a novel neurotrophic tyrosine kinase receptor type 1 gene mutation: A case report
Source: Front Med (Lausanne). 2022 Sep 7;9:955929. doi: 10.3389/fmed.2022.955929 (PMC9489851; doi:10.3389/fmed.2022.955929)

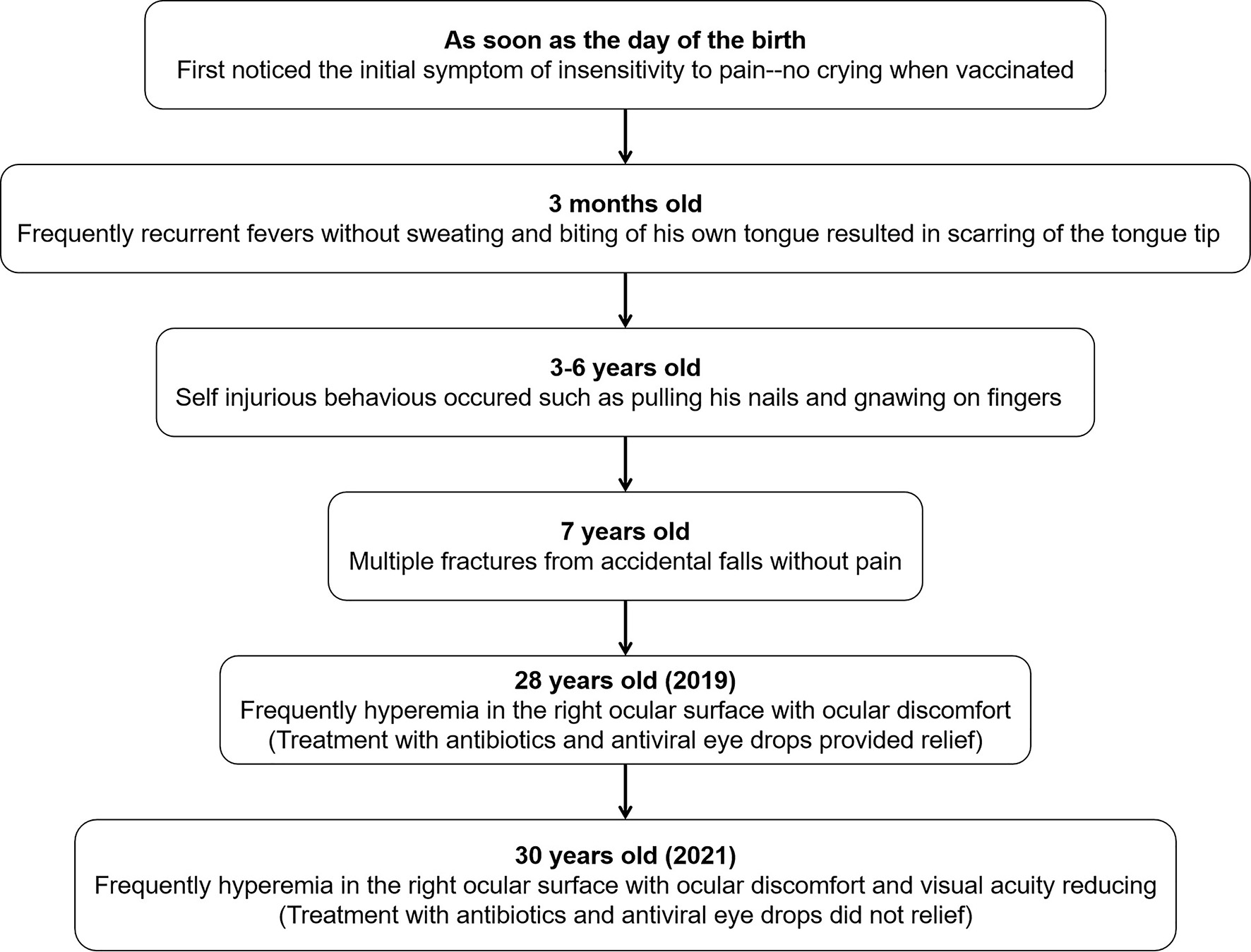

Supplement: Supplementary Figure 1 — The timeline of thepatient. [file Image_1.JPEG]
